# Meaning-making and coping in youth with chronic pain: A palliative and cultural perspective from the Philippines

**DOI:** 10.1080/24740527.2025.2496678

**Published:** 2025-05-12

**Authors:** Jeff Clyde G. Corpuz

**Affiliations:** Department of Theology and Religious Education, De La Salle University, Manila, Philippines

To the Editor,

The recently published systematic review on the coping experiences of youth living with chronic pain is both timely and critically important.^1^ As a palliative care advocate and professor in the Philippines for the past decade, I have witnessed firsthand how chronic pain, especially among the youth, often remains an invisible and underaddressed burden within public health and education systems.^2^ This study contributes significantly to our understanding of how young people adapt, suffer, and strive for meaning while living with chronic pain.

The synthesis of two key themes – “A Different Way of Being” and “Learning to Get By” – captures the complex psychosocial and existential challenges that youth face.^1^ Pain, in this context, is not merely a physiological symptom but a deeply disruptive force that alters one’s identity, daily rhythms, and social interactions.^3^ From my own classroom and community-based engagements in urban and rural parts of the Philippines, I have encountered students who silently endure chronic pain due to juvenile arthritis, sickle cell disease, fibromyalgia, or postinfectious syndromes. These young people often withdraw from school, experience stigma, and develop a fractured sense of self, echoing the theme of “a different way of being.”

The second theme – “learning to get by” – resonates with Filipino youth who must navigate pain amidst limited access to specialized healthcare and social support. Coping strategies in such contexts often include self-reliance, prayer, folk remedies, and digital communities, underscoring both resilience and systemic gaps.^4^ Although youth in the Global North may have better access to pain clinics and psychological support, Filipino youth frequently rely on their families, teachers, and church communities for affirmation and care.

This research invites further contextualization within the framework of palliative care, which, contrary to common misconception, is not limited to end-of-life scenarios but is fundamentally about improving quality of life in the face of chronic illness or suffering.^4^ A palliative approach to youth living with chronic pain would promote holistic assessment, anticipatory guidance, and integrative interventions including counseling, peer support, and spiritual care. Moreover, schools can serve as vital spaces for pain literacy, where teachers are trained to recognize signs of distress and make appropriate referrals.^4^

As a theology professor, I am particularly struck by the spiritual dimension of coping that remains underexplored in the reviewed studies. Filipino youth often derive meaning and endurance from their faith, perceiving suffering not only as a limitation but sometimes as a form of vocation or redemptive experience. Such theological resources, if engaged critically and compassionately, can enhance psychosocial support programs and help youth make sense of their pain.

In conclusion, I commend the authors for this nuanced synthesis. I urge health professionals, educators, and policymakers, especially in low- and middle-income countries such as the Philippines – to recognize chronic pain in youth as a public health concern requiring interdisciplinary, compassionate, and culturally sensitive responses.
